# Object Extraction in Cluttered Environments via a P300-Based IFCE

**DOI:** 10.1155/2017/5468208

**Published:** 2017-06-27

**Authors:** Xiaoqian Mao, Wei Li, Huidong He, Bin Xian, Ming Zeng, Huihui Zhou, Linwei Niu, Genshe Chen

**Affiliations:** ^1^School of Electrical Engineering and Automation, Tianjin University, Tianjin 300072, China; ^2^Department of Computer & Electrical Engineering and Computer Science, California State University, Bakersfield, CA 93311, USA; ^3^State Key Laboratory of Robotics, Shenyang Institute of Automation, Shenyang, Liaoning 110016, China; ^4^Shenzhen Institutes of Advanced Technology, Chinese Academy of Sciences, Shenzhen 518055, Guangdong, China; ^5^Department of Math and Computer Science, West Virginia State University, 5000 Fairlawn Ave, Institute, WV 25112, USA; ^6^Intelligent Fusion Technology, Inc., Germantown, MD 20876, USA

## Abstract

One of the fundamental issues for robot navigation is to extract an object of interest from an image. The biggest challenges for extracting objects of interest are how to use a machine to model the objects in which a human is interested and extract them quickly and reliably under varying illumination conditions. This article develops a novel method for segmenting an object of interest in a cluttered environment by combining a P300-based brain computer interface (BCI) and an improved fuzzy color extractor (IFCE). The induced P300 potential identifies the corresponding region of interest and obtains the target of interest for the IFCE. The classification results not only represent the human mind but also deliver the associated seed pixel and fuzzy parameters to extract the specific objects in which the human is interested. Then, the IFCE is used to extract the corresponding objects. The results show that the IFCE delivers better performance than the BP network or the traditional FCE. The use of a P300-based IFCE provides a reliable solution for assisting a computer in identifying an object of interest within images taken under varying illumination intensities.

## 1. Introduction

One of the primary color segmentation tasks is to extract objects (regions) of interest from an image, since a variety of vision-based applications rely on the quality of the extracted objects. Over the past years, many researchers have used color segmentation algorithms to extract regions of interest, but the low robustness of the existing algorithms to illumination variation in cluttered environments is still problematic. For example, Felzenszwalb and Huttenlocher defined a predicate for measuring the evidence for a boundary between two regions using a graph-based representation of the color image. The developed algorithm constructs the boundary of the graph by comparing the difference between two components and their internal differences, respectively, computed by minimum spanning tree (MST) and the edge weights based on the absolute intensity difference or all three (red, green, and blue) of the color plane segmentations for color images [1]. Dony and Wesolkowski introduced an edge detection approach for color images, which was based on the calculation of the vector angle between two adjacent pixels. The method detected only chromatic differences so that it was suitable for applications where differences in illumination were irrelevant [2]. Shi and Malik treated color image segmentation as a graph-partitioning problem and proposed a global criterion, that is, the normalized cut, for segmenting the graph. The normalized-cut criterion measures the total dissimilarity between the different groups as well as the total similarity within the groups. Then, they optimized this criterion using a computational approach based on the generalized-eigenvalue technique [3]. Malik et al. used contour and texture analysis for color image segmentation. They provided an algorithm for partitioning greyscale images into disjoint regions of coherent brightness and texture. The cues of contour and texture differences were exploited simultaneously. Then, they introduced a gating operator based on the texturedness of the neighborhood of a pixel that facilitates cue combination. Finally, the spectral-graph theoretical framework of normalized cuts was used to find partitions of the image in regions of coherent texture and brightness [4]. Albalooshi and Asari proposed a self-organizing lattice Boltzmann active-contour (SOLBAC) approach for segmentation while preserving the precise details of the object's region of interest. Even though the approach improved the computational time cost, the computer could not effectively identify the object of interest [5]. In terms of the psychology of object and pattern recognition, Brewer and Williams think that the pattern or object recognition is the process by which the brain recognizes light, shapes, and colors as particular objects or patterns. It is the process of “assigning meaning to the visual input by identifying the objects in the visual field.” This ability combines perception, attention, and memory [6]. Therefore, it is too difficult to identify an arbitrary object depending only on machine understanding, even if it seems simple for humans, since the recognition process is so sophisticated.

General segmentation algorithms usually process the entire image instead of the regions of interest [7]. However, these segmentation algorithms suffer from a computational time that is too long to satisfy the real-time requirements because a number of segments that are not of interest have to be processed when the algorithms are applied to vision-based robot-tracking systems. For robot operations, the segmentation of only regions of interest to fulfill robot tasks in real time should be a priority. For example, the objects of interest are goals, robots, doors, and so forth, in RoboCup [8]. For a match between two robot teams, the use of fast and robust algorithms for extracting these objects is the key step to winning the match. The popular algorithms proposed for this goal were mostly the color-based segmentation and geometrical image-processing methods [9]. Among them, the most commonly used color space is HSV [10] and the most commonly used geometrical image-processing tool is the Hough transform for circle detection [11, 12]. Moreover, Kaufmann et al. proposed the visual robot-detection technique by training the vertical and horizontal color histograms and other features using two BP networks and combining the output of the two neural networks to make the final classification decision [13]. For extracting an object of interest in cluttered environments under diverse and varying light illumination for robot navigation or operations, the robustness and fast processing time of the algorithms are critical.

In this paper, we present a method of segmenting regions (objects) of interest with color similarity via the P300-based IFCE. First, we design a 3X3 P300 paradigm to stimulate the targets, that is, the interesting-object candidates, including their corresponding fuzzy parameters and seed pixels. An object of interest to which a subject pays attention is identified while the P300 stimuli are flashing. Second, to extract an object of interest reliably, the IFCE proposed herein processes each detected pixel based on the angle between two vectors from the detected pixel to the seed pixel in RGB space coordinates. The vector length corresponds to the pixel illumination intensity and the direction corresponds to the color. The IFCE is much more robust than other color-extraction algorithms because the pixel illumination intensity and the color are separately represented. This algorithm extracts an object of interest quickly since the corresponding seed pixel and fuzzy parameters are predetermined in the training process. Last, we conducted comparative studies of the proposed algorithm with a BP network and the traditional FCE. The results show that the proposed P300-IFCE method is very robust for segmenting regions of interest. This study not only benefits people with severe motor disabilities, but also works as an auxiliary means of assisting the nondisabled man when both of his hands are busy, for example, our current project on control of an underwater manipulator via brainwaves [14]. In this application, the operator uses a P300 paradigm to control the underwater manipulator, while both the operator's hands are used to operate the underwater vehicle movement.

The motivations of this study are trying to address the following issues: First, effectively extracting an object of interest in a cluttered environment usually suffers from the following: how to use a computer to model the object like human understanding in mind and how to extract the object of interest from the cluttered environment under varying illumination conditions. In order to solve these two issues, we propose the method for object extraction via the P300-based IFCE. The P300 paradigm is used to identify an object of interest for robot navigation to simplify the complex process of identifying the object using a computer. Although the P300 paradigm could not directly represent the operator's mind, it indirectly maps the operator mental activities into his/her intention to identify the object which the machine should extract, while the IFCE operator is used to deal with the varying light conditions to improve the quality of the object of interest to be extracted. Second, the trend to developing an intelligent robot system is trying to combine human and machine intelligence. The study in this paper would be an attempt to fuse brainwaves, which indirectly or directly represent the human intentions, and the fuzzy logic-based IFCE operator, which is a typical computational algorithm, to enhance the performance of the object extraction in a cluttered environment. Mental activities through brain signals in psychology may not need a complex computational process, but currently it is very difficult for engineers to use the conclusions from psychology studies to implement the process without knowing the inside principles. Finally, this study would especially benefit people with severe motor disabilities; they cannot express their mind through a keypad, a mouse, or even speaking a word. In this circumstance, the P300 paradigm, as an auxiliary tool, establishes argumentative communication for them to select an object of interest in order to express their mental activities. By analysing the P300 components from their brain signals, the machine can identify which objects of interest should be extracted with help of the IFCE algorithm.

Up to now, most of the BRI approaches have focused on low level control of a robot system via brain signals [15–21]. For example, the works [22, 23] used four or six visual stimuli designed in the SSVEP or ERP model to control a humanoid robot's walking behaviors. On the contrary, the study in this article applies the P300 paradigm into a BRI system at a high level to assist the computer to express an object of interest that a human understands. Following the introduction, Section 2 describes the IFCE in detail and how to use it to extract an object.  Section 3 gives an introduction to the P300-based seed-pixel selection, which includes establishing a P300 model, acquiring data and analysing signals, and guaranteeing that the seed pixels represent objects of interest.  Section 4 presents some experiments in cluttered environments and compares the P300-based IFCE with two other algorithms to validate the robustness and efficiency of the proposed P300-based IFCE method. In addition, the last section draws some conclusions and puts forward some ideas for future work.

## 2. Improved Fuzzy Color Extractor

The fuzzy color extractor (FCE) was first proposed as an Iterative Fuzzy-Segmentation (IFS) algorithm by Li [24]. He applied IFS to extract color components of a chemical plume and its odour source for visual confirmation of the identified odour source [25]. The fuzzy color extractor can directly extract the chemical plume and its source by defining their color patterns. However, the color patterns are defined in the RGB space and the components of R, G, and B vary due to changes in illumination intensity. Once the illumination intensity changes, the color patterns are supposed to be recalibrated. In this paper, the traditional FCE is modified by defining a new color pattern to improve its robustness under different illumination intensities. Herein, the new color pattern is defined by the angle between the vectors of two pixels in RGB space coordinates and then replaces the R, G, and B values as the input of the traditional FCE. Based on the newly defined color pattern, a pixel is classified as belonging or not belonging to the target after fuzzification and defuzzification. The IFCE will be explained in detail as follows.

### 2.1. Color Pattern Definition

**Figure 1:**
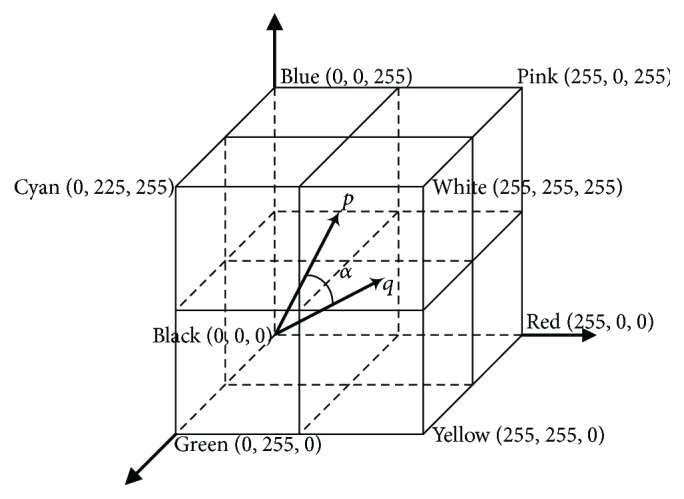
RGB space coordinate system.

Traditionally, the colors of an image are described in the RGB space, where colors are represented by their red, green, and blue components in an orthogonal Cartesian space, as shown in Figure 1. The color of each pixel *p*(*m*, *n*), denoted by (*m*, *n*)_RGB_, is processed to separate its red, green, and blue components (*p*(*m*, *n*)_R_, *p*(*m*, *n*)_G_, *p*(*m*, *n*)_B_) [7]. To distinguish between two different pixels, for example, *p*(*m*, *n*) and *q*(*s*, *t*), three variables must be calculated, as presented in (1). In this paper, a new color pattern is put forward that compresses these three variables into a single variable. First, each pixel in RGB space is regarded as a three-dimensional vector from the original point to this pixel, as shown by points *p* and *q* in Figure 1. The length of the vector represents the illumination intensity while the direction of the vector represents the color. Thus, the illumination intensity and the color of a pixel are decomposed so that the representation method is able to adapt to variations in illumination conditions. The compressed single variable is described as (2), where *d*(*p*, *q*) represents the difference between the two pixels *p* and *q*. In the new color pattern, the angle between two vectors replaces the three distances of RGB values, which reduces the influence of illumination-intensity variations:

(1) difp,qR=pm,nR−qs,tR,difp,qB=pm,nB−qs,tB,

(2) dp,q=arccos⁡pm,nR·qs,tR+pm,nG·qs,tG+pm,nB·qs,tBpm,nR2+pm,nG2+pm,nB2·qs,tR2+qs,tG2+qs,tB2,

where 0 ⩽ *m*, *s* ⩽ *M*, 0 ⩽ *n*, *t* ⩽ *N*, and *M* and *N* indicate the size of the array containing the image.

### 2.2. Fuzzy Rules

Here, we apply the following fuzzy rules to process the input *d*(*p*, *q*):

If *d*(*p*, *q*) is zero,
then *p*(*m*, *n*) and *q*(*s*, *t*) are matched.
If *d*(*p*, *q*) is negative or positive,
then *p*(*m*, *n*) and *q*(*s*, *t*) are unmatched.

In fact, *q*(*s*, *t*) often corresponds to a seed pixel, which can represent an object to be extracted. Thus, “matched” means that *p*(*m*, *n*) is matched with the seed pixel. Both rules indicate that the pixel *p*(*m*, *n*) belongs to the object to be extracted if the angle between *p*(*m*, *n*) and the seed pixel in the RGB coordinate system is small enough; otherwise, *p*(*m*, *n*) does not belong to the object.

### 2.3. Fuzzification and Defuzzification

**Figure 2:**
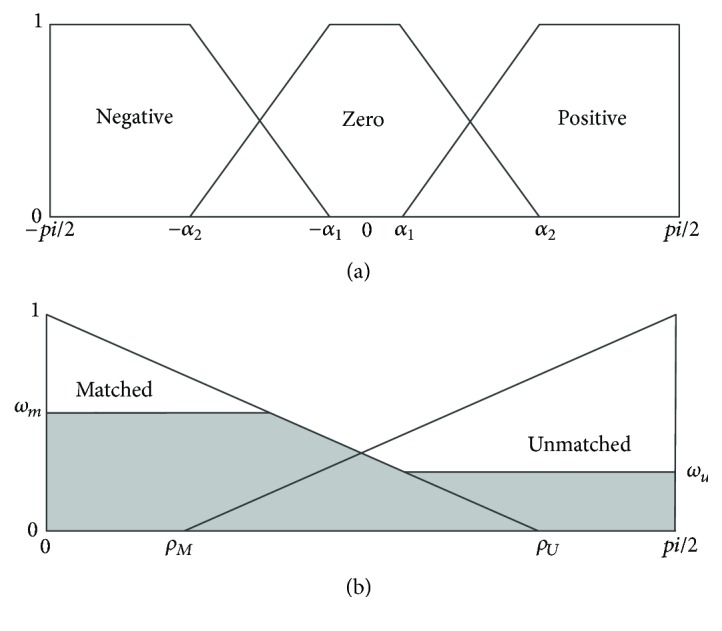
(a) Membership functions for angles. (b) Membership functions for defuzzification.

When we obtain the angle *d*(*p*, *q*), the membership can be calculated.  Figure 2(a) shows the membership functions (*μ*_*N*_(*x*), *μ*_*Z*_(*x*), *μ*_*P*_(*x*)) for the input fuzzy variables (negative, zero, and positive) that are defined by

(3) μNx=1−pi2≤x<−α2x+α1α1−α2−α2≤x<−α10−α1≤x≤pi2,μZx=0−pi2≤x<−α2x+α2α2−α1−α2≤x<−α11−α1≤x<α1α2−xα2−α1α1≤x<α20α2≤x<pi2,μPx=0−pi2≤x<−α1x−α1α2−α1α1≤x<α21α2≤x<pi2.

Figure 2(b) shows the membership functions (*μ*_*M*_(*x*), *μ*_*U*_(*x*)) for the output fuzzy variables (matched, unmatched), which are defined by

(4) μMx=ρU−xρU0≤x<ρU0ρU≤x≤pi2,μUx=00≤x<ρMx−ρMpi/2−ρMρM≤x≤pi2,

where *ρ*_*M*_ + *ρ*_*U*_ = *pi*/2. Based on the membership functions for angles *d*(*p*, *q*), the fuzzy rules produce the matched weight *ω*_*m*_ and unmatched weight *ω*_*u*_ according to

(5) ωm=μZdp,q,ωu=max⁡μNdp,q,μPdp,q.

Figure 2(b) shows the produced areas in the output domain for the case in which *ω*_*m*_ and *ω*_*u*_ cut *μ*_*M*_(*x*) and *μ*_*U*_(*x*). A crisp output value, Δ*ρ*_*F*_, is calculated by the centroid-defuzzification method, as shown in

(6) $$\begin{array}{c} \Delta \rho_{F}=\frac{\int \mu_{\text{out}}\left(x\right)x\;dx}{\int \mu_{\text{out}}\left(x\right)dx}, \end{array}$$

where *μ*_out_(*x*) represents the envelope function of the areas cut by *ω*_*m*_ and *ω*_*u*_ in the fuzzy output domain. If Δ*ρ*_*F*_ < *σ*, where *σ* is a threshold, *p*(*m*, *n*) is extracted; otherwise, *p*(*m*, *n*) is not extracted. The IFCE can be understood as a mapping operator between angle *d*(*p*, *q*) in the RGB space and a difference Δ*ρ*_*F*_ in the intensity space under a fuzzy metric.

### 2.4. Subregion Generation and Object Extraction

Given a seed pixel, similar pixels that are “matched” in one image are extracted. However, the extracted pixels may include some discrete points. Not only the object itself but also some noise is extracted. Usually, the noise is distributed discretely and randomly, so some measures must be taken to ignore it. Meanwhile, the pixels belonging to some subregions need to be merged together in order to extract the entire object.

**Figure 3:**
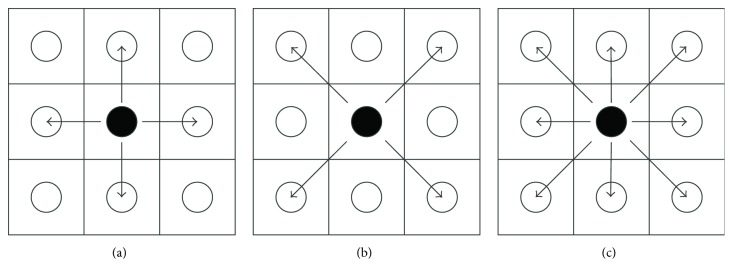
The subregion growing towards (a) its 4 adjacent neighbors, (b) its 4 diagonal neighbors, and (c) its 8 surrounding neighbors.

Here, we use a technique that is different from the traditional region-growing based method [26] to generate subregions. The subregion generation, as shown in Figure 3, occurs together with the “matching” process. First, the first seed pixel representing the object is obtained from the stimulus target induced by the P300 potential. Second, the angles between the seed pixel and every other pixel in the image are calculated and the minimal angle is selected as the starting pixel. Third, we use the IFCE to determine the “matched” pixels in one subimage based on the pattern, as shown in Figure 3(c). When all the “matched” pixels based on the seed pixel are determined in a subregion, the subregion extraction is complete. Then, a new pixel is selected with the minimal angle from the remaining pixels and the process described above is repeated to extract the next subregion. The extraction process continues until (7) is not satisfied or there is no “matched” pixel adjacent to the subregion:

(7) $$\begin{array}{c} d\left(\;p,q\;\right)=\sum_{i}{\left[\;p{\left(\;m,n\;\right)}_{i}-q{\left(\;s,t\;\right)}_{i}\;\right]}^{2}<r, \end{array}$$

where *i* represents the R, G, and B values and *r* is the threshold that was preset based on experience. *d*(*p*, *q*) calculates the distance between one pixel and the seed pixel in RGB space. The equation guarantees that the pixel to be extracted is near to the seed pixel in order to extract the pixel whose color is similar to the seed pixel. Still, this threshold may change based on different colors, but not the image. The threshold remains the same for objects with similar colors.

Lastly, several subregions are obtained. In this paper, it is assumed that the subregion with the largest number of pixels is the object. During the process of pixel extraction, there might be some bad pixels belonging to the object that are not extracted to the subregion because of the reflection of light. Therefore, we fill in the pixels missing in the object. When the number of unextracted pixels between two extracted pixels is smaller than a given threshold, the unextracted pixels should be regarded as part of the object and be extracted. This process is executed in every row and column. As a result, only the subregion representing the object is extracted.

## 3. Seed Pixels and Fuzzy Parameters

This paper proposes a method for object extraction from a cluttered image, which combines the P300 paradigm and IFCE. Just as with the most object recognition methods [27, 28], the proposed method consists of offline and online phases. The offline phase is to establish a dataset of the parameters of objects in a cluttered image for the IFCE, including seed pixels and fuzzy parameters, while the online phase is to use the P300 paradigm to select an object of interest, that is, to select its corresponding parameters from the dataset for the IFCE-based object extraction process.

**Figure 4:**
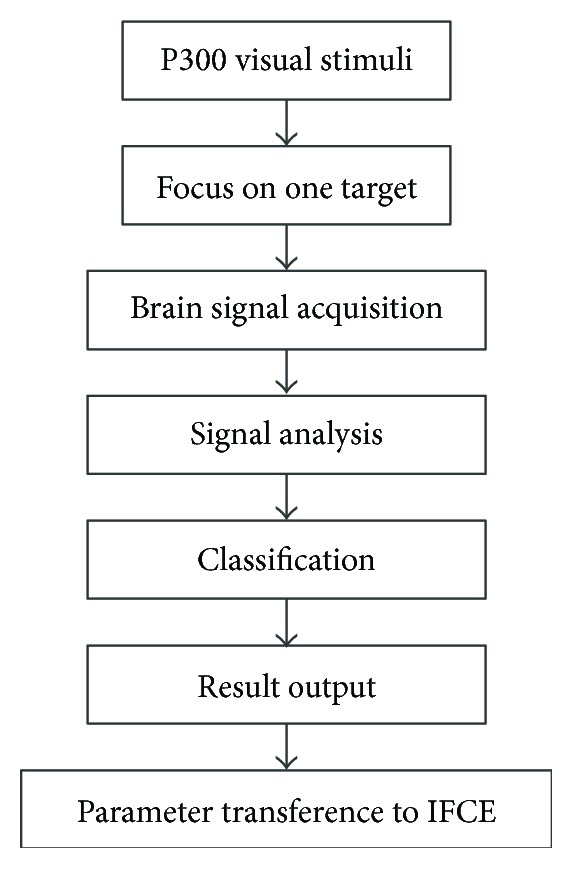
The process of P300-based seed-pixel selection.

Figure 4 shows the selection process of P300-based object parameters. During this process, the subject first focuses on an object of interest represented by the P300 visual stimuli. Then, his/her brain signals acquired by the EEG device are analysed and classified to choose the parameters of the object of interest from the dataset. Last, the corresponding parameters are delivered to IFCE to extract the object on which the subject is focusing. The detailed explanation of the process is addressed in the following paragraphs.

### 3.1. P300 Paradigm

**Figure 5:**
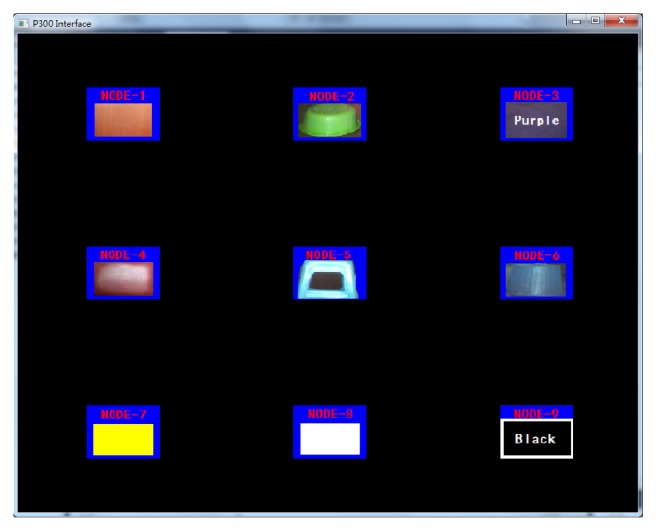
3 × 3 P300 speller user interface.

Among various EEG models, P300 has the advantages of having multiple targets, high accuracy, and short training time. Therefore, we designed a 3 × 3 P300 speller as the user interface, as shown in Figure 5, to represent an object of interest that contains the RGB-value information of the seed pixel and the fuzzy parameters. Once the target, that is, the object of interest, is selected, the corresponding parameters are delivered to the IFCE to extract the object in an image. Therefore, the accuracy and real-time performance of the P300 model can directly influence the performance of the IFCE. In this article, we need 7 targets and reserve 2 additional targets to expand the number of seed pixels when needed.

**Figure 6:**
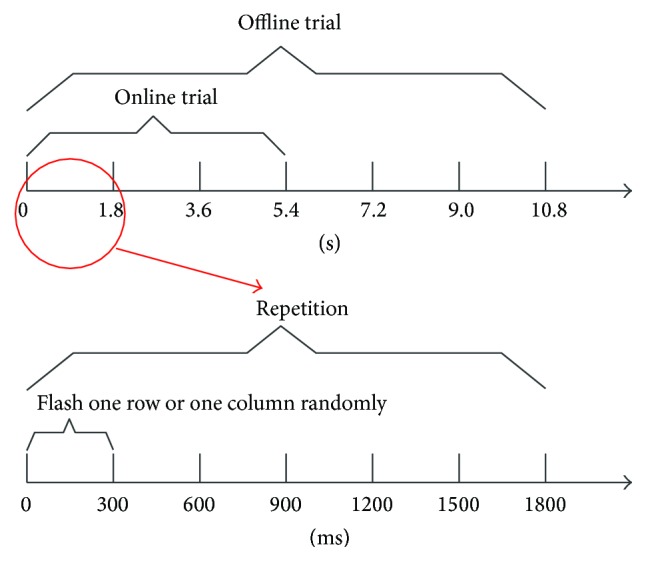
The time sequence of the offline trial, online trial, one repetition, and one flash.

The P300 experiment consists of offline training and an online experiment. The offline training is used to train a classifier for the online experiment. During a P300 experiment, one repetition consists of flashing each of the six rows and columns one by one in a random order. The presentation time of a row or a column is 200 ms and the interstimulus interval (ISI) [29] is 300 ms; thus one display cycle (one repetition) is 1.8 s. A single repetition flashes every row and every column once, and every target flashes twice. A number of repetitions constitute a trial, in which the subject is asked to focus on only one target. Each target consists of 6 repetitions in the offline training process and 3 repetitions in the online experiment. In this article, each target flashes 6 times (namely, 3 trials) before the P300 model outputs a result. The subject is suggested to count the times where the target is presented [30].  Figure 6 shows the time sequence of the offline trial, online trial, one repetition, and one flash.

EEG signals were recorded from 8 subjects who participated in 6 sessions of the P300-model testing, including 3 offline and 3 online experiments. The acquired neural signals are amplified, preprocessed by an analog low-pass filter of 50 Hz, and digitalized with a sampling frequency of 1000 Hz. The standard EEG cap with 30 channels is used to acquire the EEG signals.

### 3.2. Signal Analysis

Signal analysis consists of preprocessing, feature extraction, and classification. In preprocessing, a digital band-pass filter with a bandwidth of 0.1 to 30 Hz filtered the data segment lasting 800 ms from a stimulus appearance. Then, we selected data from 50 ms to 800 ms to remove the average component. Lastly, data after 50-fold frequency reduction were used to form the feature vectors. In feature extraction, the data of 30 channels were connected together head to tail. Thus, the feature vectors of the target and nontarget were obtained. For classification, we used a Fisher Linear Discriminant Analysis (FLDA) as our classifier. The two classes of target and nontarget were marked with Labels 1 and −1, respectively. The FLDA classifier in the online experiment used the classifier trained by offline data.

### 3.3. Seed-Pixel and Fuzzy-Parameter Selection

A seed-pixel dataset consists of the RGB values of every pixel and the corresponding fuzzy parameters, as shown in Table 1. O1 to O7 represent seven objects in cluttered environments, corresponding to the first seven stimuli in the P300 model. The RGB value is obtained from the original image shot by a camera. For each group of R, G, and B values, a pixel belonging to the object is randomly extracted, except for some reflective pixels. The remaining parameters in Table 1 are obtained according to the results of the fuzzy training process based on experience. Once one target of the seven is selected, the corresponding parameters will be delivered to the IFCE and the object of interest is to be extracted.

Thus, the subject can select a seed pixel from a predefined set of pixels to directly identify different targets. Not only the seed pixel but also the fuzzy parameters are optimized choices because they are all pretrained before the experiment. Since the seed-pixel selection is straightforward, the computation time of seed-pixel determination is greatly reduced and the real-time performance of the system is improved.

## 4. Experiments and Results

### 4.1. Evaluation of the P300-Based Model

The evaluation of the P300-based model consists of two parts: offline training and online experiment. In the offline training process, the acquired data that are processed and classified as described in the previous section are used to train the FLDA classifiers of different subjects. In the online experiments, we applied the FLDA classifiers to recognize the target and provide feedback to the subjects in real time.

Eight subjects (seven right-handed, one left-handed) with normal vision volunteered to undergo the experiments. The collected neural signals are divided into the training and testing data for the FLDA classifier. To evaluate the P300-based model objectively, the acquired data are randomly chosen to train the FLDA classifier, and then the remaining data are used as test data for testing the FLDA classifier. The evaluation process repeats the procedure for training and testing the FLDA classifier 6 times.  Table 2 lists the accuracy rates of the classification results for every time and every subject.

After the offline training, in the online experiment, the FLDA classifier with the highest accuracy from 6 evaluations of every subject were chosen. However, in the online experiment, we finalized a classification result after voting based on 3 repetitions. The target that has more than two votes will be selected as the final target. Similarly, Table 3 lists the accuracy rates of the classification results for every time and every subject in the online experiment. We conducted the online experiment 3 times and each experiment includes 9 targets for 2 cycles. Then, the accuracy rates are summarized for every experiment.

By analysing the evaluation results, we find that the accuracy rates of the offline and online experiments are higher than 95%. Furthermore, some subjects, despite having no experience with the P300-based model, can achieve an accuracy rate of 100%. The results demonstrate that the P300-based model is very suitable for seed-pixel selection.

### 4.2. Segmentation of Objects of Interest

To demonstrate the advantages of the IFCE, we contrast the segmentation results obtained using a BP network, the traditional FCE, and the IFCE. The object recognition results will be demonstrated in the following part.

**Figure 7:**
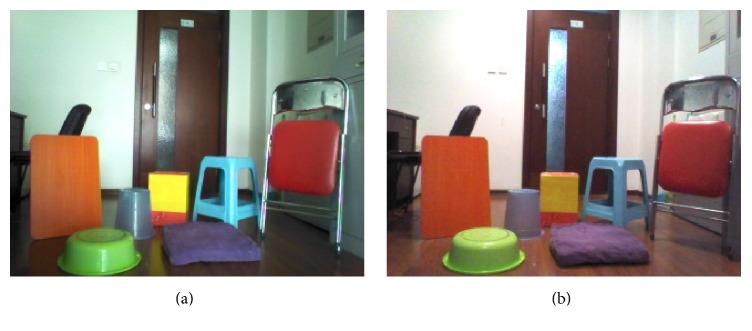
The original images. (a) Early in the morning with sunlight. (b) Late in the evening with the lights on.

**Figure 8:**
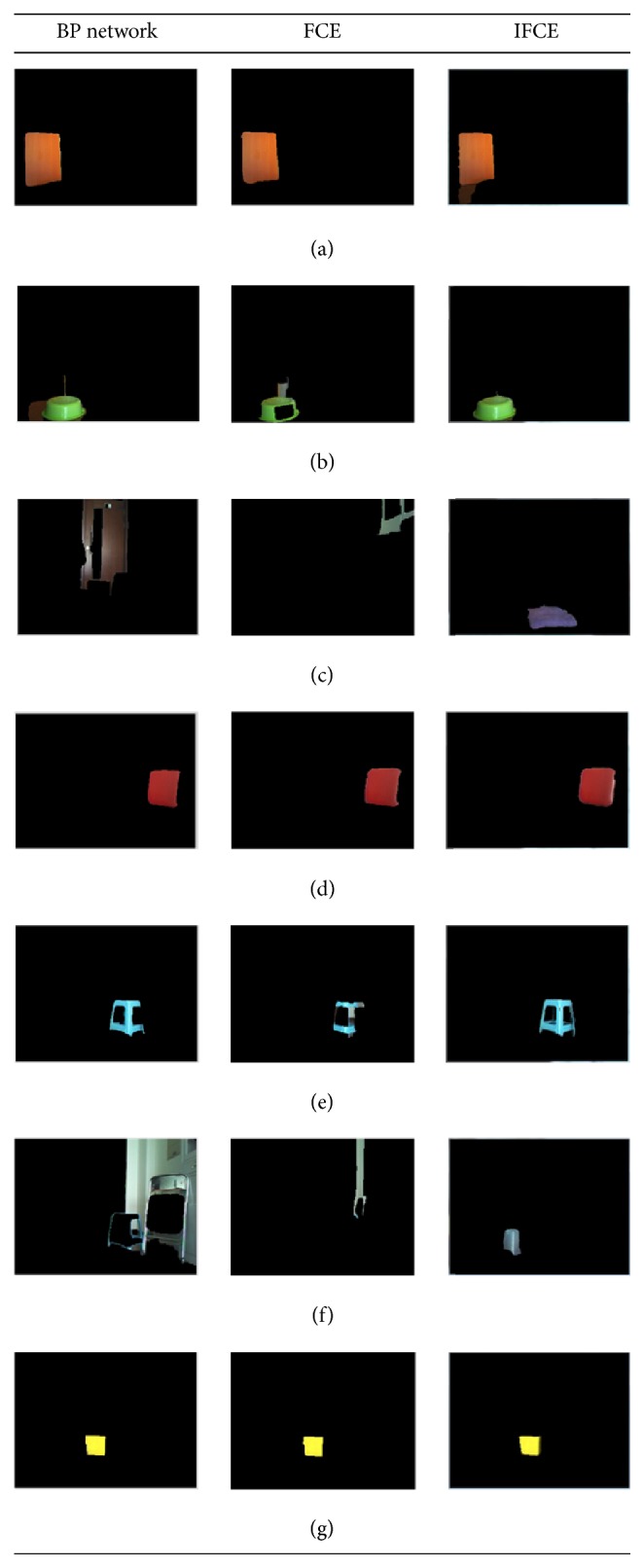
Comparison of the results obtained using a BP network, FCE, and IFCE (early in the morning with sunlight).

**Figure 9:**
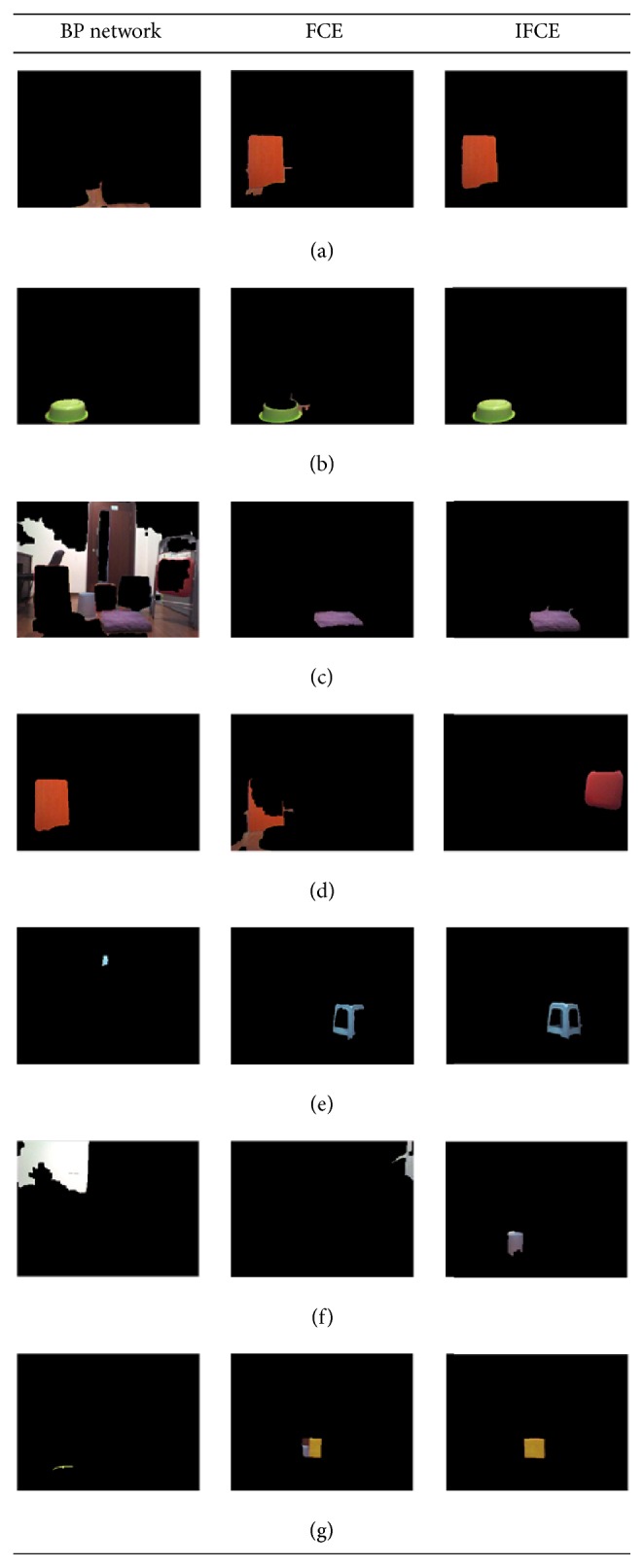
Comparison of the results obtained using a BP network, FCE, and IFCE (late in the evening with the lights on).

Figures 7(a) and 7(b) show two original images taken by a NAO robot [31] whose camera is set to a resolution of 320 × 240 pixels. The objects shown in the image are all based on the material from the camera itself. However, the image is not dependent on the property of the camera so any camera that can take color pictures should work. The images reveal an ordinary scene in daily life and the illumination intensity varies from early in the morning with sunlight to late in the evening with the lights on. The color threshold methods [32] cannot be used to segment the objects from the image because there are too many colors in the image. Therefore, this paper uses a BP network [13] and the traditional FCE to segment the images in order to contrast the results with those of the IFCE. Figures 8 and 9 show the segmentation results of 7 objects obtained using a BP network, the FCE and the IFCE. Additionally, the BP network and FCE also used the subregion-generation method in the process of segmentation.

When the illumination condition is early in the morning with sunlight, the results shown in Figures 8(a), 8(d), and 8(g) indicate that the BP network and FCE worked with the bright colors and achieved good performance. For some dark colors, Figures 8(b) and 8(e) show that the BP network and the FCE basically extracted the objects, but the details of the objects were not revealed well and some noise pixels were also extracted along with the objects. Furthermore, in Figures 8(c) and 8(f), the objects of interest were totally submerged in the noise. However, the proposed IFCE was able to successfully extract all 7 of the objects of interest. When the illumination intensity changes late in the evening with the lights on, the results in Figure 9 show the strong robustness of the IFCE, in contrast with the BP network and FCE. As is shown in Figures 9(a), 9(b), 9(c), 9(e), and 9(g), the objects of interest were extracted, but the two methods performed badly in terms of presentation of the details and noise elimination. Figures 9(d) and 9(f) show that the wrong regions were extracted as the objects of interest. Note that Figures 8 and 9 share the same parameters even though the illumination intensities are different. Thus, the IFCE was more robust than the BP network and FCE. For Figures 8(f) and 9(f), the two parameters of the IFCE are changed to obtain the segmentation result. In total, the IFCE is able to adapt to illumination-intensity variations without recalibrating the parameters, but the BP network possibly needs to be retrained when the illumination intensity changes. In our experiment, we used the IFCE to segment the image and acquire the objects of interest.

### 4.3. Discussion

To illustrate the illumination-intensity variations, we compare the saturation and the lightness between the 7 objects in the two images.  Table 4 lists the variation rates of the same pixel under different illumination intensities. As the table shows, the IFCE is able to segment the regions of interest for saturation-variation rates ranging from 9.1% to 57.2% and lightness-variation rates ranging from 4.4% to 33.3%. Therefore, the IFCE is adaptive to a wide range of illumination-intensity variations. For the object in Figures 8(f) and 9(f), the difficulty in segmenting is caused not only by a relatively large illumination-intensity variation but also by its small areas; thus, a recalibration process is still needed to obtain the object.

Furthermore, the training processes of the parameters of the BP network and IFCE (similar to the traditional FCE) are different from each other. The BP network often needs a variety of samples to obtain a good model, while the IFCE only needs one pixel belonging to the object to train the fuzzy parameters. Therefore, the BP network needs to extract as many pixels belonging to the object as possible in order to obtain enough training samples. An interest region covering a small area may be not enough to train a BP network. For any region of interest, a single seed pixel is able to train the fuzzy parameters for segmentation. Thus, the IFCE has more potential to reduce the burden of humans and computers.

In terms of seed-pixel selection, it is the first key step for image segmentation. In this paper, the first very initial seed pixel is manually selected by using a mouse to click a pixel on the object of interest and the corresponding fuzzy parameters are preset. During the offline phase of the setting process, the remaining seed pixels for extracting this object are automatically determined based on (7). The criterion for evaluating good performance is defined by observing object contours as whole as possible and redundant pixels as few as possible. This strategy for the seed-pixel selection process combining the manual selection of the initial seed pixel and automatic determination of the remaining seed pixels delivers better performance than only using manual selection of all the seed pixels. The P300-based model directly selects a seed pixel and its corresponding fuzzy parameters from a preestablished dataset that has no accidental error with the help of the P300-based model. In addition, the introduction of brain signals provides an effective means to assist the computer in recognizing the objects that are of interest to humans.

For any given experimental environments, we analyse those objects in an image needed for specific operation tasks to establish a dataset for the P300 paradigm. In this paper, we usually choose the objects that are commonly used for robot navigation tasks, so the proposed method works as long as the objects exist in the environment since each object has the same parameters even under different conditions. The dataset grows when more objects in an unknown environment needed to be extracted.

The reasons for applying the P300 paradigm into object recognition in the BRI area are as follows: First, it is an unsolved problem: how to use a machine to effectively represent objects of interest that a human understands. Like controlling robot motion via brainwaves, applying the P300 paradigm is indirectly incorporating human intentions to identify the object of interest for robot navigation, which is able to provide abundant visual stimuli to expand objects of interest in complex environments and to achieve high recognition accuracy because they represent human intentions correctly. Second, the visual stimuli are directly presented in the form of objects of interest, instead of simple words or squares, because the objects of interest might provide subjects with more instinctive information to understand the visual stimuli meaning and help subjects concentrate on their mental activities [33], which elite the high quality P300 potentials. Finally, the P300-based IFCE would be the first attempt to combine the BCI technologies with machine vision, which may lead to fusing human knowledge and machine intelligence.

## 5. Conclusions

In this article, we integrate the EEG-based P300 model into the object extraction process. The extraction of an object is a complex process from the aspect of psychology, so it is very difficult for a computer to understand an object of interest without human involvement. Therefore, this article draws support from the P300-based model to assist the computer in extracting an object of interest. Herein, the P300-based paradigm was used to induce a stimulus target representing an object of interest including its seed pixel in the image. Once the seed pixel was obtained via P300 brain signals, the segmentation method uses the IFCE to process the image to generate the subregions of interest that form the object of interest from the image.

To validate the feasibility of the system, we conducted some experiments and compared the results obtained using the proposed segmentation method with those obtained using other methods. Eight subjects participated in the P300 offline and online experiments and the average accuracy rates reached higher than 95%. Each target of the P300 interface represented a seed pixel containing the corresponding RGB values and fuzzy parameters of the object. After the target was locked by the subject, the data would be transferred to the IFCE for segmentation. At last, the IFCE was tested on two images taken in a daily life environment with different illumination intensities. The results showed that the IFCE had a better performance than the BP network and the traditional FCE, especially for some objects with dark colors. Moreover, there is no need to recalibrate the fuzzy parameters of the IFCE even when the illumination intensity changes. Therefore, even if the image changes as a whole, the method is still effective as long as the objects in the original image appear in a new image. If a new object appears in the image, the corresponding parameters are obtained to update the dataset by a short time training process which then can be used to extract the object.

Due to the robustness and precision of interesting-object extraction, the exact color and shape information can be revealed vividly, which provides an effective means for automatically identifying an object of interest by matching a property with the object via P300 brain signals. Once an object of interest is identified, the NAO is able to find a path to approach the object and to conduct the operation [34]. Nevertheless, the current IFCE algorithm aims at extracting an object with “single” similar color. As for a very complex object, we can consider combining the color information with others, such as object shapes and textures, to represent the object. In addition, we try to set multiple seed pixels representing an object with “multiple” similar colors to extract them via IFCE. Then, these multiple similar colors near to each other will merge together to form the object. Generally, each object may have its specific colors different from the others, so using these specific colors may be the very convenient way to solve the problem.

Our future work will focus on applying the P300 paradigm into robot vision because processing images acquired from the camera of a robot provides a variety of applications in daily life. Combining the P300-based paradigm with the IFCE will make robot operations more effective and efficient to serve in complex environments and especially provide an auxiliary means for people who are unable to use both hands in some circumstances. Furthermore, this is our first attempt to combine the brain signal with the objects extraction algorithm. In the future, first we will develop algorithms to automatically update the dataset when any untrained object appears, and second we will apply an optimization algorithm, such as a generic algorithm (GA), to determine the initial seed pixel and fuzzy sets instead of manual adjusting.

## Figures and Tables

**Table 1:** Seed-pixel dataset (partial).

Object	RGB	*α*_1_	*α*_2_	*ρ*_*M*_	*σ*
O1	186,69,34	.2	.3	.2	.5
O2	104,140,53	.2	.3	.2	.5
O3	130,100,142	.1	.2	.3	.6
O4	141,47,48	.1	.4	.3	.6
O5	87,133,166	.1	.3	.3	.6
O6	134,147,181	.15	.25	.8	.5
O7	176,134,36	.2	.3	.4	.4

**Table 2:** Classification accuracy rates of the offline training.

Subject	Acc. 1	2	3	4	5	6	Average
S1	94.44	100	100	100	94.44	100	98.15
S2	100	100	100	100	100	100	100
S3	100	100	100	100	100	100	100
S4	94.44	100	100	100	100	100	99.07
S5	100	100	100	100	94.44	100	99.07
S6	100	100	91.67	100	91.67	100	94.44
S7	100	100	88.89	100	88.89	100	96.30
S8	83.33	100	91.67	100	100	100	95.83

**Table 3:** Classification accuracy rates of the online experiment.

Subject	Acc. 1	2	3	Average
S1	100	100	100	100
S2	100	100	100	100
S3	100	100	100	100
S4	94.44	100	100	98.15
S5	100	100	100	100
S6	100	100	100	100
S7	100	94.44	94.44	96.29
S8	100	100	100	100

**Table 4:** Variations in the saturation and lightness at different illumination intensities.

Object	Saturation		Variation rate (%)	Lightness		Variation rate (%)
O1	140	163	16.4	120	103	14.2
O2	102	92	9.8	121	96	20.7
O3	44	40	9.1	90	120	33.3
O4	144	128	11.1	96	88	8.3
O5	173	74	57.2	145	118	18.6
O6	32	16	50.0	114	109	4.4
O7	240	157	34.6	150	101	32.7
